# Environmental performance of waste electrical and electronic equipment plastic recycling based on life cycle assessment

**DOI:** 10.1177/0734242X261451602

**Published:** 2026-06-03

**Authors:** Mohammad Naji Nassajfar, Jouni Havukainen, Elaheh Aryapour, Mariam Abdulkareem, Mika Horttanainen

**Affiliations:** 1 Department of Sustainability Science, School of Energy Systems, Lappeenranta-Lahti University of Technology, Finland

**Keywords:** waste electrical and electronic equipment, plastic recycling, chemical recycling, mechanical recycling, life cycle assessment

## Abstract

There is a significant global rise in the amount of waste electrical and electronic equipment (WEEE). This waste typically contains more than 20% plastic, which can have significant environmental benefits if properly treated and recycled. However, recycling plastics from WEEE is a challenging task due to the complexity of the waste composition, which consists of several polymers, many of which contain heavy metals, additives, and brominated flame retardants. This study presents both technical and environmental concerns in the resource recovery of WEEE plastics. Based on results of a prior research on the composition of waste electrical and electronic plastics (WEEP) in Finland, this study assess the environmental impacts associated with the recovery of WEEP by comparing four technically available WEEP treatment methods: energy recovery (incineration), mechanical recycling of composite plastics, mechanical recycling for separated polymers, and chemical recycling by pyrolysis. This study concludes that mechanical recycling with plastic separation has the best environmental performance among the presented scenarios for all selected impact categories except for the acidification potential impact category. The substitution ratio of virgin plastics and the efficiency of the pyrolysis process were the primary factors contributing to the environmental impacts of WEEP treatment options.

## Introduction

In 2019, the total amount of globally generated waste electrical and electronic equipment (WEEE) was about 53 Mton (Forti et al., 2020) of which—depending on the type of electronic equipment—10–50 wt% consists of plastic materials (Martinho et al., 2012). Moreover, fossil-based waste electrical and electronic plastics (WEEP) contain valuable organic materials, which from the resource point of view, can be recovered as plastic materials, secondary chemical feedstock, or fuel after undergoing proper treatment. The global volume of WEEE is increasing; at the same time, EU targets for its recycling and recovery are also becoming more stringent. The EU Directive 2012/19/EU sets goals for recovering, reusing, and recycling 10 categories of WEEE; however, these targets cannot be achieved solely through the recovery of metal and glass. Therefore, plastic must be included in the recovery or recycling processes. Recovering WEEP poses a significant challenge due to its composition, which consists of a variety of materials such as acrylonitrile–butadiene–styrene (ABS), high-impact polystyrene (HIPS), polypropylene (PP), polystyrene (PS), styrene-acrylonitrile (SAN), polyesters, polyurethane (PU), polyamide (PA), blends of polycarbonate (PC)/ABS, and blends of HIPS/poly(p-phenylene oxide; Vilaplana and Karlsson, 2008). Furthermore, various additives, some of which are hazardous substances, can change the properties such as color, melting point, flammability, and density. These additives may be flame retardants and various stabilizers or plasticizers (Dimitrakakis et al., 2009; Erickson and Kaley, 2011; Schlummer et al., 2007).

Despite advancements in plastic recycling, the plastic fraction of electronic waste is not consistently addressed. The metal fraction of WEEE holds significant economic value, leading to the separation of metals using manual or automatic methods. However, the remaining plastic fraction, hindered by flame retardants and regulatory requirements, cannot be treated alongside plastics from other sources. Consequently, it is often directed to incineration plants for energy recovery or exported to other regions for further treatment. The treatment choices for WEEP include incineration, mechanical recycling, and chemical recycling. Some of these recovery options are already available in the market, whereas others will be technically feasible in the near future (Arena and Ardolino, 2022). Mechanical recycling of WEEP involves collecting, sorting, and processing plastic fractions of WEEE into new products without changing their chemical composition. This process aims to recover plastic materials through methods such as shredding, melting, and molding. Chemical recycling corresponds to procedures that chemically degrade plastic waste to produce materials with high purity the same as the original monomers or feedstock. Ultimately, energy recovery takes place, which unlike the previous methods does not preserve the feedstock, but harvests the embedded chemical energy of materials through incineration in waste-to-energy (WtE) plants.

There are several published life cycle assessment (LCA) studies of plastics in WEEE, particularly examining the pyrolysis of WEEP. Alston et al. (2011) compared the environmental impacts of WEEP pyrolysis with the alternative options of mechanical recycling, incineration, and landfill and concluded that incineration is the most effective method of reducing carbon deposits and effectively reducing landfill space but has the highest impact on climate change. However, the pyrolysis option supports resource conservation. Pyrolysis or incineration preserves the majority of resources, with the exact savings varying based on the source of electricity that the incineration supplants. Arena and Ardolino (2022) discussed methods for plastic waste valorization, proposing a classification of both traditional and innovative treatments. Their focus was on solutions suitable for managing challenging plastics waste from WEEE, end-of-life vehicles, and construction and demolition waste. Regarding light and not-brominated WEEP, they concluded mechanical recycling has a lower global warming potential (GWP) than energy recovery by combustion or disposal in sanitary landfills.

Ardolino et al. (2021) conducted a study in which they evaluated four management scenarios for WEEP. They compared the use of current options available in Europe with a combination of innovative options (ideal scenarios) and evaluated the negative consequences of improper options currently being used for WEEP shipped to less developed regions (real scenarios). The study found that this new approach would largely improve the environmental performance of WEEP treatment.

Other studies (Ardolino et al., 2021, Arena and Ardolino, 2022) on WEEP have pursued the idea that the WEEP fractions can be separated via mechanical recovery and then a series of treatment methods can be based on the composition of waste fractions. This approach demonstrates promising environmental benefits; however, the technology which is based on selectively dissolving a specific polymer for the mechanical recovery scenario is still in the developmental phase, and since this technology incorporates an extended recycling chain, the overall efficiency of recycling might be decreased.

The present research was conducted as a part of the “PLASTin” project (Plastin, 2022), and the contribution of this article is to assess the environmental impacts of possible treatment practices for WEEP in Finland using the technical details provided by the research partners, including data about the waste composition and sorting procedures. To address the case-specific characteristics such as the composition of WEEP and the source of energy, there was a need to conduct an LCA study. This study assesses the environmental impacts of four WEEP recovery options namely, energy recovery (incineration), mechanical recovery of composite plastics and separated plastics, and chemical recovery (pyrolysis). The data utilized regarding the composition of WEEP was derived by Hamod (2021) and Parkar (2021) in the PLASTin project. The measurements were performed on six specimens of mixed WEEP consisting of ABS, PC/ABS, PS, and unidentified plastics. The sample comprised various e-waste plastics, ground into dimensions ranging from 40 to 76 mm. These recovery options are either currently available or available in the near future. The share of bromine (Br)-rich plastics is based on separation tests carried out in the PLASTin project realized between 2019 and 2022.

## Materials and methods

The LCA study follows the ISO 14040/44 guidelines, which is a well-established method used to evaluate the environmental impacts of products and services throughout their life cycle (SFS-EN ISO 14040, 2006; SFS-EN ISO 14044, 2006). The LCA comprises four steps (a) goal and scope definition, (b) life cycle inventory, (c) life cycle impact assessment, and (d) interpretation.

### Goal and scope definition

The goal of this study is to assess the environmental impacts of WEEP recovery using an LCA developed in accordance with ISO 14040/14044 standards. GaBi software by Sphera version 10.6 was used for the LCA. Employing a process-based approach, this study considers impacts directly linked to the WEEP system. The analyzed WEEP management scenarios consider only the plastics obtained by the dismantling of WEEE officially collected in Finland. Local sources and industry data from Finland (Kuusakoski Oy) have been used. The composition of WEEP was determined by using a near-infrared (NIR) device. NIR is a material classification technology which works based on absorption of electromagnetic radiation. A hand-held NIR device can identify different plastic materials and detect hazardous elements in products (Pasquini, 2018). However, due to the inefficiency of the device, some of the WEEP fraction is not identifiable and is assumed to have the same composition as the rest of the waste. The environmental assessment of WEEP recovery was carried out by examining four scenarios: energy recovery, mechanical recycling of composite plastics, mechanical recycling for separated polymers, and chemical recycling by pyrolysis. The results are intended to support decision-making by evaluating the potential environmental impacts of WEEP treatment, thereby contributing to improved environmental performance in WEEP management. The functional unit of this research is set as the treatment of 1 Mton of WEEP, free of metals.

#### System boundaries

**Figure 1.**
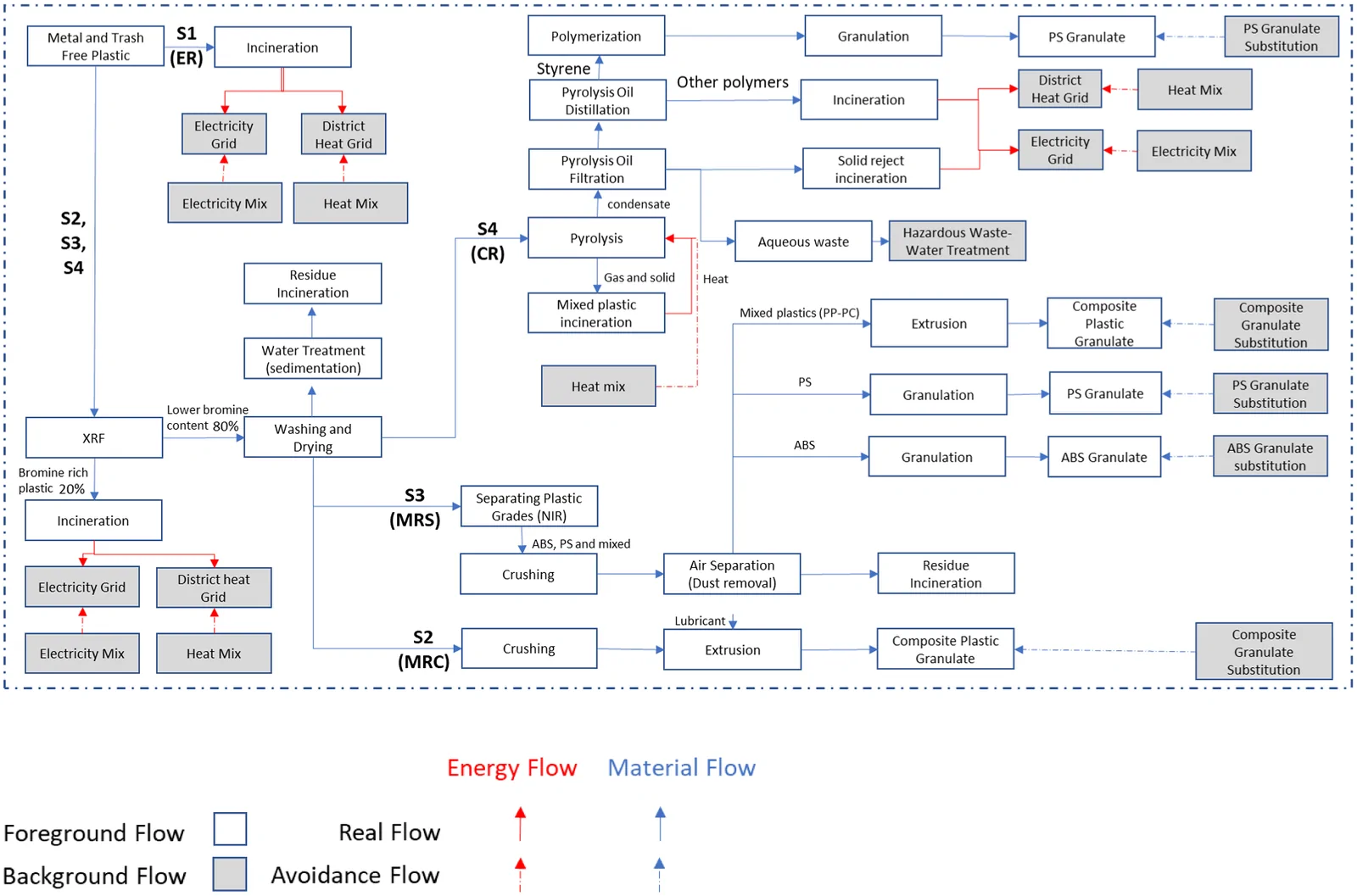
An overview of the four scenarios in the LCA study; S1-ER; S2-MRC; S3-MRS; S4-CR. LCA: life cycle assessment; S1-ER: scenario 1, energy recovery; S2-MRC: scenario 2, mechanical recycling composite plastic; S3-MRS: scenario 3, mechanical recycling separation; S4-CR: scenario 4, chemical recovery.

The system boundaries in Figure 1 show a “gate-to-gate” analysis, encompassing the collection and dismantling of mixed plastic WEEE as the input stage at the initial gate, and recycled plastics and energy as the output stage at the final gate. The initial feedstock is metal and trash-free WEEP (from which the metals and waste such as fine fractions containing mixed materials have been removed);therefore, neither the separation of metals from the plastic fraction of WEEE nor the treatment for metal fractions are considered. The system boundary also includes the avoided emissions obtained by replacing the materials and energy from the presented waste treatment options. The system expansion method was applied, using a credit system to take into consideration materials and energy recovered from the WEEP treatment. Credit here refers to avoiding environmental impacts by replacing virgin materials with recycled materials and replacing energy from the grid with recovered energy in the WEEP treatment processes. The avoided energy and material in Figure 1 are shown with dotted arrows (avoidance flow) and the real WEEP and energy consumption is demonstrated with normal straight lines (real flow). The transportation of materials to the waste handling facility is excluded from the system boundaries since it is assumed to be similar for all presented scenarios. Supplementary functions needed to run the primary processes, such as providing electricity and thermal energy from the grid mix, were included in the background systems.

#### Scenario description

This study considers four scenarios as listed in Table 1.

**Table 1.** Description of scenarios of life cycle assessment.

Perspective	Scenario	Description
Incineration with energy recovery	S1-ER	All WEEP to incineration
Mechanical recovery (1) (composite plastics)	S2-MRC	Mechanical recovery-composite plastic granulates
Mechanical recovery (2) (separated plastics)	S3-MRS	Mechanical recovery-NIR separation of ABS, PS, PP-PC
Chemical recycling	S4-CR	Chemical recycling using pyrolysis; styrene is selected as the main product of CR since 27% of pyrolysis oil is built from styrene

ABS: acrylonitrile–butadiene–styrene; PC: polycarbonate; PP: polypropylene; PS: polystyrene; NIR: near-infrared; WEEP: waste electrical and electronic plastics.

In the baseline scenario (S1-ER), WEEP is directed to an incineration plant, as this is the most common treatment method, generating energy that substitutes for heat and electricity production based on the average Finnish energy mix.

In the second scenario (S2-MRC), composite production from mixed plastic is included in the study because it allows for a high yield of products, but it also results in lower-quality material that can only substitute limited applications. However, in the third scenario (S3-MRS), the plastic fractions of PS, ABS, and PP-PC are separated by NIR technology, which provides better-grade plastic polymers to substitute virgin plastics. The fourth scenario (S4-CR) explores the chemical treatment of WEEP using a pyrolysis process. The product of pyrolysis is a high-quality oil that can be used as high-purity feedstock to substitute virgin plastics in the plastic production industry.

The electricity from the grid is assumed to be the electricity production of Finland in 2021 (nuclear 35%, hydro 28%, wind 13%, biomass 2%, natural gas 7%, hard coal 4%, and solar 1%). Thermal energy production in Finland for the same year involved biomass 54%, coal 15%, heavy fuel oil 4%, light fuel oil 1%, natural gas 14%, and peat 12%.

In addition to energy substitution, the chemicals produced in the chemical recycling and NIR separation routes can replace virgin oil-based plastics. However, as indicated by the system boundary, recycling WEEE using mechanical or chemical treatment first requires the removal of brominated flame retardants from the WEEE fraction using X-ray fluorescence (XRF) technology. XRF utilizes X-rays to stimulate the atoms within a substance, generating a distinct fluorescence light emission that aids in the identification of different elements present. This method enables the detection of Br, with the literature reporting detection limits of 300 parts per million (ppm) for portable devices (Aldrian et al., 2015).

By applying XRF, 20% of incoming WEEP identified as Br-rich plastic is sent for incineration (Kärki et al., 2022). Thus, 200 kg of the functional unit is directed to WtE, whereas the remaining 800 kg (with low Br content) undergoes chemical or mechanical recycling.

### Inventory data

In this study, the source of the inventory data consisted of scientific literature, interviews, and comments from researchers working on WEEE recycling and experts in a WEEE recycling company. The data for substituted energy as well as virgin plastic granulates were obtained from the Ecoinvent and Sphera databases.

As a part of this project, Kärki et al. (2022) reported the amount of the Br-free and Br-rich plastic in experimental trials as listed in Table 2. The corresponding value for unidentified plastics is divided based on the mass fraction of each polymer from the known proportion of the WEEP. In all four scenarios, the Br-rich fraction of WEEP is treated in a hazardous waste incineration plant and produces electricity and heat which substitutes the electricity and heat from the grid.

**Table 2.** Share of measured (Hamod, 2021) and adjusted mass fractions of plastic polymers in WEEP, with the unidentified fraction proportionally allocated based on identified polymer masses.

Bromine free				Bromine rich		
Plastic fraction	Measured fraction (NIR identified) (%)	Allocated unidentified fraction (%)	Total adjusted fraction (%)	Measured fraction (NIR identified) (%)	Allocated unidentified fraction (%)	Total adjusted fraction (%)
ABS	28	22	50	25	33	58
PP	9	7	16	5	7	12
PS	10	8	18	6	8	14
PC	10	8	18	7	9	16
Unidentified	44	—	—	57	—	—
Total	100	—	100	100	—	100

ABS: acrylonitrile–butadiene–styrene; PC: polycarbonate; PP: polypropylene; PS: polystyrene; NIR: near-infrared; WEEP: waste electrical and electronic plastics.

#### Background processes

The inventory data for energy carriers such as fuel, electricity, and heat were obtained from the GaBi database as well as the required data for all other upstream and downstream raw materials and processes.

#### Avoided impacts

The study employed a system expansion approach to avoid allocation issues, identifying the avoided impacts associated with products displaced by market obtained co-products and incorporating these substitutions into the model. Substituting virgin-grade polymers with recycled polymers on a one-to-one basis is inappropriate due to the typically inferior technical properties of recycled plastics. The substitution approach follows the framework suggested by Vadenbo et al. (2017), which evaluates the substitution ratio (SR) of an existing market product (virgin plastics) with a secondary material (recycled plastics) based on four parameters:

(1) SR=U×η×α×π

The potential physical amount of the secondary resource ( U ) is the amount of each waste fraction in S3-MRS. The associated recovery efficiency of each resource ( η ) depends on the selected scenario and consists of the sorting and process efficiency for handling WEEP. The substitutability ( α ) indicates the functionality offered by the recovered resource in comparison to the conventional resource. The market response ( π ) represents the proportion of the secondary resource capable of replacing the available product on the market.

Table 3 outlines the utilized parameters to calculate the SR value. The sorting efficiencies of the polymers were obtained from Cardamone et al. (2021) since these values were not measured in the PLASTin project. The process efficiency is assumed to be 95% for all polymers since it is the average value reported by De Meester et al. (2019) and Cardamone et al. (2021). Evaluation of the substitutability of each polymer follows the methodology presented by Rigamonti et al. (2020) and Vadenbo et al. (2017), involving the identification of key technical parameters and consideration of corresponding values for both secondary and virgin polymers. For all the plastic fractions, impact strength values determined by Parkar (2021) in the PLASTin project were utilized. Values are calculated for 1 ton of polymer sent for mechanical recycling.

**Table 3.** Assumed values for the quantification of the SR of plastic products.

Parameter	ABS	PS	PC	PP
Potential physical amount (U), ton	1	1	1	1
Recovery efficiency (η), %	64	67	91	89
Sorting efficiency, %	68	70	96	94
Process efficiency, %	95	95	95	95
Substitution-ability (α), %	100	89	49	75
Market response (π), %	100	100	100	100
SR of virgin polymer, %	64	59	45	67
The potential amount of the secondary resource (S3-MRS) per FU, kg	394	134	120	120
Avoided production of virgin polymers (MRS) per FU, kg	254	79	53	80

Sorting efficiency obtained from Cardamone et al. (2021). The substitutability values were calculated by identifying key technical parameters and considering corresponding values for both secondary and virgin polymers. Scenario S1-ER: ER by incineration.

ABS: acrylonitrile–butadiene–styrene; ER: energy recovery; MRS: mechanical recycling separation; NIR: near-infrared; PC: polycarbonate; PP: polypropylene; PS: polystyrene; SR: substitution ratio.

In the incineration scenario S1-ER, all the WEEP fractions without any treatment are transported to the incineration plant. The waste plastic incineration datasets from the Sphera database represent the incineration of ABS, PC, PP, and PS with a net calorific value of 39, 30, 45, and 41 MJ/kg, respectively.

WtE plants in Europe employ incineration as a thermal treatment method for municipal solid waste. These plants adhere to legal requirements by incorporating dry flue gas cleaning and selective catalytic reduction to remove nitrogen oxides. The environmental impacts of waste collection, transportation, and any pre-treatment processes are not included in the data provided. The modeled plant comprises an incineration line equipped with a grate and a steam boiler.

#### Scenario S2-MRC: Mechanical recycling-composite plastic

The XRF process separates WEEP based on the Br content into two fractions of Br-free and Br-rich. The fraction with lower Br content accounts for 80% of the processed WEEP.

In scenario S2-MRC, the WEEP fraction with a low Br content is crushed and sent for plastic extrusion, and lastly undergoes a compression molding process. In modeling scenario S2-MRC, pre-set processes from the GaBi database were utilized, such as electricity production, extrusion, and incineration. The process for incineration of the Br-rich fraction was the same as the incineration in scenario S1-ER. The data for the washing/drying and crushing processes were obtained from the literature and by gathering data from plastic recycling companies. Supplemental Table S2-1 presents the detailed parameters for scenario S2-MRC.

Mechanical recycling results in a decline in polymer quality after each cycle, ultimately reaching a stage where the resin can no longer be mechanically recycled (Schyns and Shaver, 2021). This implies that the result should be more accurately described as a “down-cycled” material, which has limited potential for entering the market. Therefore, to achieve a realistic scenario, the recycled composite plastic originating from WEEP is not a suitable replacement for virgin plastic; however, recycled WEEP material could replace recycled composite plastics from other resources.

**Figure 2.**
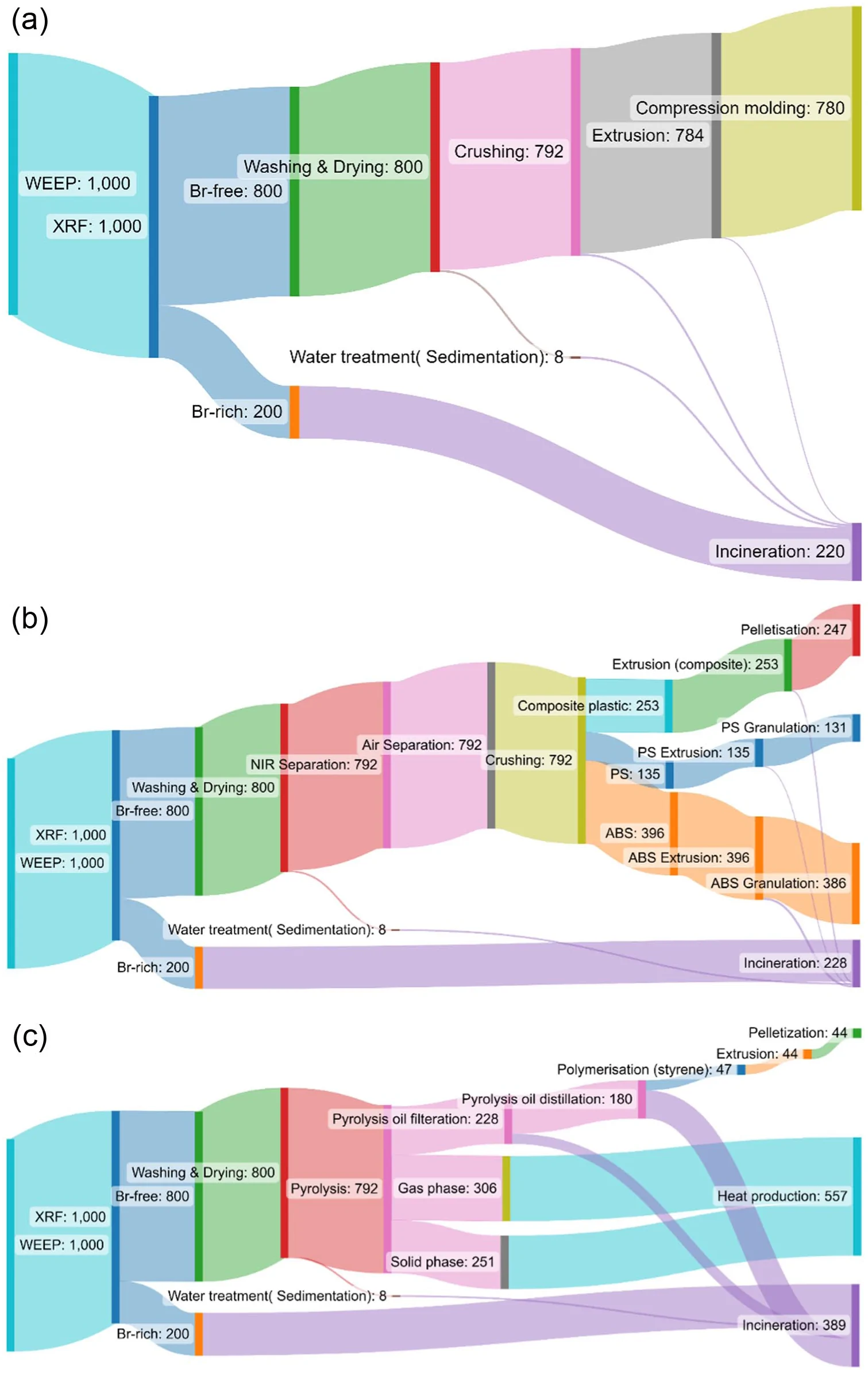
From up to down: the mass flow of scenarios S2-MRC (a), S3-MRS (b), and S4-CR (c). S2-MRC: scenario 2, mechanical recycling composite plastic; S3-MRS: scenario 3, mechanical recycling separation; S4-CR: scenario 4, chemical recovery.

Figure 2a demonstrates the mass flow of WEEP in scenario S2-MRC. From 1 ton of WEEP, 780 kg is received for compression molding, and is a composite plastic suitable for low-grade applications. The remaining 220 kg of the WEEP which consist of the Br-rich fraction (200 kg) and residues from washing, crushing and filtration processes (20 kg) are subsequently incinerated.

#### Scenario S3-MRS: Mechanical recycling of separated polymers

In scenario S3-MRS, different polymers from the low-Br WEEP fraction are separated using NIR technology. Subsequently, the separated polymer fractions are crushed into small pieces (they need to be in larger pieces for good separation in the NIR). To remove the dust from the fraction, it is processed using air separation, with the dust residue being sent for incineration. In scenario S3-MRS, the ABS fraction undergoes a similar treatment process as the PS polymers. ABS and PS fractions are converted to granulates via a granulation process.

During the granulation phase, the polymer fraction undergoes pelletization and extrusion, while any plastic impurities are directed to incineration. The processing route for the (PP-PC) fraction is similar to the composite plastics treatment in scenario S2-MRC.

Figure 2b shows the mass flow of WEEP in scenario S3-MRS. From 1 ton of WEEP, 764 kg (386 kg ABS, 247 kg mixed plastic, and 131 kg PS) is converted into plastic granulates. The rest of the waste is separated through XRF, washing, and filtration processes and subsequently, the WEEP reject fractions are incinerated.

#### Scenario S4-CR: Chemical recycling via pyrolysis

For this scenario, inventory data for the pyrolysis scenario is mainly derived from Rieger et al. (2021). After washing and crushing, WEEP is fed into a pyrolysis reactor where the plastic fraction undergoes thermochemical treatment and decomposition at 650°C. The reactor employs a specially designed system combining internal and external heating, raising the temperature of the medium to 650℃ to prevent flame retardants from forming polyhalogenated dioxins and furans. The pyrolysis reactor decomposes the plastics into a gaseous and vaporous state leaving the reactor through a cyclone and condensation train where eventually the product forms three phases of oil (condensate), gas, and solids. The remaining gas and solids are subsequently burned on-site to maintain the required thermal energy for the pyrolysis process (Rieger et al., 2021).

The condensate contains solids and aqueous phase materials. Prior to further treatment of the pyrolysis oil, the solids and aqueous phase of the crude condensate are filtered, and the filtrates are separated. Subsequently, the pretreated oil (condensate) is fed into the fractional distillation apparatus. The fractional distillation column initially evaporates the oil and then gradually cools the vapor down in a controlled manner. Based to the boiling points of benzene, toluene, ethylbenzene, xylene, and phenolic compounds present in the processed oil, distillate fractions comprising high concentrations of substances such as styrene, alpha-methyl styrene, phenol, and cresols are separated. Supplemental Table S4-1 lists the inventory data for the scenario S4-CR.

Figure 2c demonstrates the mass flow of WEEP in scenario S4-CR. From 1 ton of WEEP, only 44 kg is converted to PS granulates. The rest of the waste is separated through XRF, washing, and as by-products of the pyrolysis process, and subsequently, the reject fractions of WEEP are incinerated. The recovered energy in the incineration process serves as a substitute for heat and electricity that would otherwise be generated from alternative sources, such as fossil fuels. The utilization of recovered energy from incineration can result in a reduction in the waste volume and mitigation of environmental impacts associated with energy production.

### Impact assessment

For the impact assessment, this study employed the CML 2001–January 2016 methodology, which is widely recognized and utilized in LCA studies. The CML method enables the evaluation of environmental impacts across various impact categories (Rigon et al., 2019). Human toxicity potential-inhalation factor (HTP inf) was incorporated, since recycling WEEE can lead to the release of heavy metals into the environment (Rosenbaum et al., 2008). The “Abiotic resource depletion potential-fossil” impact category was covered since plastic is conventionally manufactured from fossil oil. The GWP is included due to global importance, and plastic production generates significant greenhouse gas (GHG) emissions. Acidification is an important impact category when the incineration process is involved; moreover, oil refining emits Sulfur oxide emissions, which are precursors to acid rain (Posch et al., 2008, Seppälä et al., 2006).

#### Sensitivity analysis

Sensitivity analyses are employed to assess how variations in data and methodological decisions influence the outcomes of an LCA study (SFS-EN ISO 14040, 2006). Two primary reasons for performing a sensitivity analysis are: (1) to pinpoint which parameters most significantly affect the system and how they vary under different conditions and (2) to analyze how uncertainty in a model’s output can be traced back to various sources of uncertainty in the model’s input (Guo and Murphy, 2012). Th sensitivity analysis in this research was carried out to determine how the system behaves under different conditions. Three parameters were assessed for their sensitivity and effect on the environmental impacts: changing the SR of granulates, substituting wood with composite plastics in S2-MRC, changing the oil yield of pyrolysis in S4-CR.

As a part of system expansion, the recycled plastic products in scenarios S2-MRC and S3-MRS substitute recycled plastics from other resources (baseline of S2-MRC) or virgin plastics (S3-MRS). The quality of produced WEEP granulates is potentially lower than the quality of granulates from other sources. Therefore, a substitution parameter was utilized to compensate for the lower quality of WEEP. The value for SR varies for different polymers and different grades of granulates. Therefore, as a sensitivity analysis parameter, scenario S2-MRC evaluates the use of virgin plastic alongside the SRs shown in Table 3, as well as an alternative case assuming uniform SR values of 50%. Table 4 presents three sensitivity assumptions: variation in the SR value of WEEP granulates in scenarios S2-MRC and S3-MRS, substitution of wooden bars with WEEP in scenario S2-MRC, and variation in the pyrolysis oil yield in scenario S4-CR.

**Table 4.** Cases for sensitivity analysis.

Uncertainty in S2-MRC (SR of Recycled Plastics (RP))	
Wood_100%	WEEP substitutes wood at a ratio of 100%
BL_RP_100%	BL, WEEP substitutes RP at a ratio of 100%
VP_Table 3	WEEP substitutes VP with SRs presented in Table 3 for each plastic fraction
RP_50%	WEEP substitutes recycled plastics (RP) at an SR of 50%
Uncertainty in S3-MRS (SR of recycled plastics)	
VP_100%	WEEP substitutes VP at an SR of 100%
BL_VP_Table 3	Baseline, WEEP substitutes VP at SRs presented in Table 3 for each plastic fraction
VP_50%	WEEP substitutes VP at an SR of 50%
Uncertainty in S4-CR (Yield of pyrolysis process)	
BL_POY_29%	Baseline, Pyrolysis Oil Yield (POY) is 29%
POY_91%	POY is 91%

In S2-MRC and S3-MRS, the substituted materials or ratios are varied. In S4-CR, there are two assumptions for the yield of the pyrolysis process.

BL: baseline; POY: pyrolysis oil yield; RP: recycled plastics; VP: virgin plastics; WEEP: waste electrical and electronic plastics; SR: substitution ratio.

## Results and discussion

**Figure 3.**
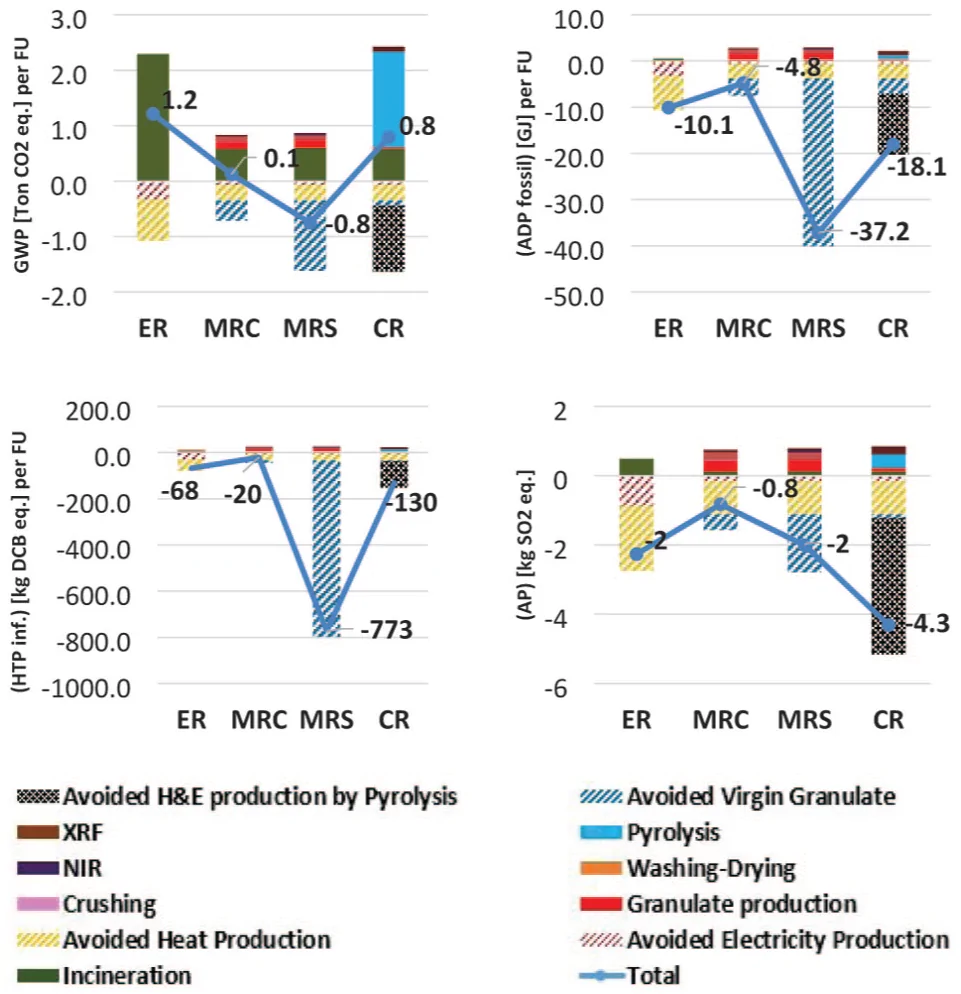
Environmental impacts of four scenarios. Global warming potential (GWP)[ton CO_2_-eq], abiotic depletion potential (ADP fossil)[GJ], human toxicity potential (HTP inf.)[kg DCBeq.], acidification potential (AP)[kg SO_2_ eq.].

Figure 3 demonstrates the environmental impacts of four studied scenarios. Mechanical recycling of separated plastics S3-MRS has the best environmental performance among the presented scenarios for all selected impact categories except for the acidification potential impact category. This is a result of the savings regarding the environmental loads that stem from substituting virgin plastic granulates with WEEE-originated plastic as well as substituting heat and electricity production with the excess heat and electricity in recycling processes.

The net GWP impact of scenario S1-ER is about 1200 kg CO_2_ eq. per ton of WEEP. The incineration of the ABS fraction has the highest contribution to the GWP of scenario S1-ER. This could be due to the high share of ABS in WEEP (more than 50%) and plastic incineration produces more than 2 tons of CO_2_ eq. per ton of WEEP.

In scenario S2-MRC, the net GWP is 0.2 ton CO_2_ eq. per ton of WEEP in a system consisting of WEEP treatment, plastics production, and energy production. The total GWP of the S2-MRC scenario excluding avoided emissions is 0.9 ton CO_2_ eq. per ton of WEEP, from which 0.6 ton CO_2_ eq. stems from the incineration of un-recycled fractions.

In scenario S3-MRS the total GHG emissions amount to −0.7 ton CO_2_ eq. per ton of WEEP. Avoiding virgin plastic production by substituting virgin plastics with recycled plastics saves 1.3 ton CO_2_ eq, which consequently offsets the CO_2_ emissions.

In scenario S4-CR, the total GHG emissions amount to 0.8 ton CO_2_ eq. per ton of WEEP. After the pyrolysis process, incineration of Br-rich plastic has the highest contribution to the GWP with 1.7 and 0.6 ton CO_2_ eq. per ton of WEEP, respectively. The energy generated from incinerating the char and gas in chemical recycling is used as the energy source to power the pyrolysis process. The total heat required for pyrolysis was determined to be 2330 MJ per ton of WEEP (Ardolino et al., 2021). However, in this study, the heat generated from incinerating the pyrolysis gas and char is calculated to be 18,700 MJ per ton of WEEP. Therefore, 16,400 MJ per ton of WEEP in excess heat is produced in the pyrolysis process, which substitutes the heat production from the grid mix. The substituted heat and electricity consequently prevent the emissions of about 1.2 ton CO_2_ eq. in the GWP impact category.

The abiotic depletion potential (ADP) fossil impact category highlights the potential depletion of fossil-oil resources. Thus, any use of fossil oil, whether as feedstock for plastic production or as an energy source, increases the ADP fossil impact category. Conversely, avoiding virgin plastic production or burning oil for heat reduces the impact in this category. In scenario S3-MRS, where separated WEEP production avoids using virgin granulates for plastic production, the ADP fossil impact category has a lower value than the other scenarios. In the baseline of scenario S2-MRC, where WEEP avoids using recycled plastics, its impact on fossil-oil consumption is minimal. In the S4-CR and S1-ER scenarios, most WEEP is utilized as an energy resource, substituting fossil fuels in the national energy mix.

The HTP impact category assesses potential human health effects linked to the release of toxic substances into the environment. It gauges the potential toxicity of a chemical to humans, considering its inhaled toxicity and the likelihood of human exposure (Rosenbaum et al., 2008). Plastic production is a significant source of toxic substances, including hazardous air pollutants and persistent organic pollutants. Therefore, in scenario S3-MRS, where WEEP replaces virgin-grade plastics, the reduction in the HTP impact category is notably higher than in other processes. Additionally, burning WEEP for energy can release toxic substances such as heavy metals, volatile organic compounds, and polycyclic aromatic hydrocarbons. As a result, in scenario S1-ER, where all WEEP is incinerated, the HTP impact is higher compared to other scenarios.

The acidification potential impact category in the LCA gauges the potential of an activity to contribute to acid rain or acidification of soil and water. Emissions of SO_2_ and NO_x_ from industrial processes and energy production are factors affecting the acidification potential in the LCA (Rosenbaum et al., 2018). Among these, energy production, especially heat production, has a substantial impact on the acidification potential category. In scenarios S1-ER and S4-CR, heat production stands out as the primary contributor to mitigating potential acidification impacts. In scenario S4-CR, heat production is mainly attributed to incinerating the Br-rich fraction and excess heat generated in the pyrolysis process. Conversely, in scenario S1-ER, where all the excess heat from pyrolysis is utilized for heat production, it has a significantly higher acidification potential impact than electricity production in Finland.

Arena and Ardolino (2022) and Cardamone et al. (2021) considered using a dissolution process to separate plastic waste that contains additives and impurities from other polymers or materials. The aim is to use a specific solvent to selectively dissolve the desired polymer. The ideal solvent for this method would be one that can selectively dissolve the targeted polymer while leaving all other polymers undissolved. Solvent-based recycling is still under development, and it is not yet widely used. On the other hand, this article proposes the Br-free fraction of WEEP can be used as a source of composite plastic (S2-MRC). Recycled WEEP composite serves as a substitute material for recycled plastic composites and is relevant where low-quality plastic is sufficient for the application requirements. This approach is considerable specifically when the WEEP is composed of several fractions and the detection and separation of polymers are not feasible.

Alston et al. (2011) assessed the environmental impacts of pyrolysis of WEEP with alternative options of mechanical recycling, incineration, and landfill and concluded that incineration is the most effective method of reducing carbon deposits and effectively reducing landfill space, but it has the highest climate change impact. However, the pyrolysis option acts in favor of resource-saving. Pyrolysis or incineration saved the most resources, with the balance depending on the source of electricity replaced by incineration. They concluded that a mechanical recycling scenario with the highest recycling rate and highest recycling quality has the least environmental impacts in the categories of climate change and abiotic depletion. However, with current recycling practices, it is not always possible to recycle WEEP at high quality. Additionally, the rate of substitution of virgin plastics with recycled WEEP significantly affects the environmental impacts.

The outcomes of this research are in line with Arena and Ardolino (2022), who reported 0.44 ton CO_2_ per ton of WEEP for pyrolysis and 1.6 ton CO_2_ per ton of WEEP for incineration scenarios. These are close compared to this study which are 0.8 and 1.1 ton CO_2_. For the mechanical recovery, Arena and Ardolino (2022) reported the GWP emissions for each plastic polymer. The average GWP emissions value for the WEEE composition of their study is −1.8 ton CO_2_. The corresponding value for scenario S3-MRS in this study is −0.7 ton CO_2_. The reason for the difference in this value might stem from different assumptions in the model and most importantly from different values for the SR and the composition of energy resources. Moreover, the avoided emissions of energy production in Finland are lower than the average emissions of energy production in Europe.

### Sensitivity analysis

**Figure 4.**
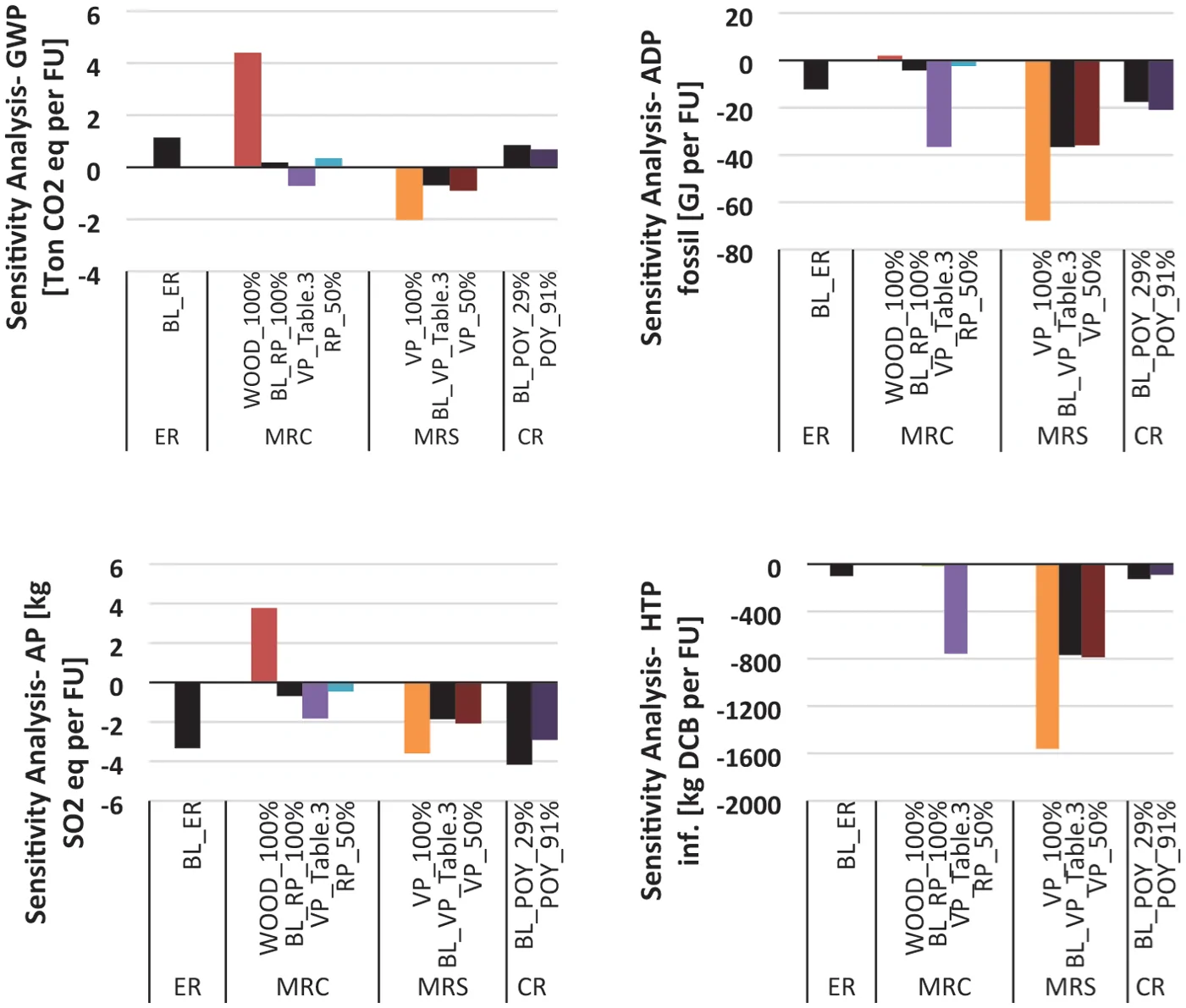
Sensitivity analysis for several substitution options in scenarios S2-MRC and S3-MRS and pyrolysis oil yields in scenario S4-CR. S2-MRC: scenario 2, mechanical recycling composite plastic; S3-MRS: scenario 3, mechanical recycling separation; S4-CR: scenario 4, chemical recovery.

The goal of conducting a sensitivity analysis is to examine how alterations of important parameters in the system affect the outcome. In this study, as shown in Table 4, three parameters were chosen for evaluation in the sensitivity analysis. The results of the sensitivity analysis are presented in Figure 4 and elaborated hereafter for each scenario.

#### Sensitivity analysis on changing the SR of granulates

In scenario S2-MRC (VP-Table 3) if WEEP substitutes virgin plastic granulates, even when implementing the SR from Table 3, the environmental impacts are lower than the baseline form of the S2-MRC scenario (BL_RP_100%). On the other hand, decreasing the SR of recycled plastics from 100% to 50% (MRC RP_50%) increases the environmental impacts of scenario S2-MRC.

In scenario S3-MRS, the recycled WEEP substitutes virgin plastic granulates. In a hypothetical situation where the practical SR of WEEP is 100% (MRS VP_100%), the environmental impacts of S3-MRS would be about 100% less than the baseline scenario. Overall, a lower SR value for virgin plastics in both mechanical recycling scenarios results in higher environmental impact categories, which is consistent with previous findings from contribution analyses of environmental impacts. Since most of the environmental benefits of scenarios S2-MRC and S3-MRS are due to the substitution of WEEP with virgin plastics, higher SR diminishes the overall environmental impacts. Another observation is that if the baseline scenario is kept for chemical recycling, then mechanical recycling options still tend to be more effective for most categories (except AP) regardless of SR values.

#### Sensitivity analysis on substituting wood with composite plastics in S2-MRC

In scenario S2-MRC, the product of the recovery process are recycled composite plastic granulates for use as the feedstock of low-grade plastic products such as pallets, outdoor plastic bars, etc. Thus, in the system expansion, the produced granulates are assumed to replace composite recycled plastic granulates from the market. Composite plastics are used for construction material purposes and there they can substitute wood for many purposes. Mixed plastic composites might not always replace virgin plastics or virgin composites because of low-grade quality for physical properties such as strength, surface structure, and color. Thus, a sensitivity analysis was conducted to compare the environmental benefit/loss of substituting wood materials with WEEE-based materials. The environmental impacts when replacing wood material with WEEE-based granulates (wood_100%) are considerably higher than the baseline results of S2-MRC (except for the HTP impact category). The reason for this was that the net GWP of wood material is assumed to be almost zero since it consists of biogenic resources and substituting wooden materials with recycled plastics causes environmental impacts. Therefore, from an environmental perspective, replacing wooden materials with recycled WEEP is not a suitable option. On the other hand, the replacement of wood is a better option than S1-ER and S4-CR if only the HTP category is taken into consideration.

#### Sensitivity analysis on changing the oil yield of pyrolysis

The yield of oil in the pyrolysis process in scenario S4-CR is potentially a sensitive parameter since it controls the amount of the final product. In the baseline scenario, this value is assumed to be 29% since Rieger et al. (2021) obtained this value in their experiments. However, other literature proposed other values. Santella et al. (2016) suggested that a higher yield of oil (91%) in pyrolysis can be achieved if the pyrolysis is carried out at 600°C and without the presence of catalysts. Therefore, as a sensitivity analysis parameter, the yield of oil from pyrolysis in scenario S4-CR was changed from 29% to 91%. When the pyrolysis yield increased from 29% to 91%, the quantity of recycled plastic produced from 1 ton of WEEP increased from 44to 140 kg, respectively. In the AP and HTP impact categories, the baseline case demonstrates fewer environmental burdens. This result is because a higher oil yield in pyrolysis means less solid and gas fractions, and in this case, the pyrolysis process acts as a net producer of heat. On the other side, a higher yield of oil in pyrolysis will result in more granulate production. However, a higher yield of pyrolysis oil here results in higher environmental impacts.

## Conclusions

This study assesses the environmental impacts of processing plastic waste from electronic waste, which is more challenging to recycle than packaging plastics, etc., due to the presence of multiple types of plastics and other materials such as Br-containing flame retardants. One limitation of the study is that the waste composition data were derived from the PLASTin project and is based solely on a Finnish case study, which may limit the generalizability of the results. Mechanical recycling of light and Br-free polymers from WEEE, such as ABS, PS, PC, and PP, can lead to significant environmental benefits. Through mechanical recycling, large amounts of CO_2_ emissions can be saved compared to energy recovery by incineration or chemical recovery.

The results of this study show that mechanical recycling with plastic separation (S3-MRS) has lower environmental impacts than incineration and chemical recycling in the environmental impact categories of GWP, ADP, and HTP.

In the mechanical recycling scenarios, the SR, which indicates the quality of the recycled plastics, has significant importance. Scenario S3-MRS had less of an environmental impact than mechanical recycling of composite material, and the quality of the produced composite granulates in scenario S2-MRC was lower than the mono-polymer granulates of scenario S3-MRS.

High-quality PS granulates can be obtained through chemical recycling; however, due to the low yield of pyrolysis, this process is not environmentally preferable when mechanical recycling is technically feasible and there is demand for products made from mechanically recycled WEEP.

The results for the S3-MRS scenario revealed that mechanically separated WEEP with the use of NIR separation technology has the potential to lower the environmental impacts in the categories of GWP, ADP, and HTP. However, for mixed WEEP, where it is not possible to separate the plastic fractions, the potential options are energy recovery (incineration), composite mechanical recovery, and chemical recovery (pyrolysis). For mixed WEEP, S2-MRC and S4-CR had lower environmental impacts than S1-ER. Several recent studies recommend the use of an innovative multi-technology waste treatment which despite good environmental potential is currently not available. As a transient solution for residual WEEP, after the mechanical recycling of separated polymers, chemical recovery and composite mechanical recovery are viable options.

## Supplemental Material

sj-pdf-1-wmr-10.1177_0734242X261451602 – Supplemental material for Environmental performance of waste electrical and electronic equipment plastic recycling based on life cycle assessment Supplemental material, sj-pdf-1-wmr-10.1177_0734242X261451602 for Environmental performance of waste electrical and electronic equipment plastic recycling based on life cycle assessment by Mohammad Naji Nassajfar, Jouni Havukainen, Elaheh Aryapour, Mariam Abdulkareem and Mika Horttanainen in Waste Management & Research
