# Supplementary material for: Environmental performance of waste electrical and electronic equipment plastic recycling based on life cycle assessment
Source: Waste Manag Res. 2026 Jun 3;44(10):1777–88. doi: 10.1177/0734242X261451602 (PMC13612888; doi:10.1177/0734242X261451602)
Supplement: Supplementary file 1 [file sj-pdf-1-wmr-10.1177_0734242X261451602.pdf]

Environmental performance of WEEE plastic recycling based on life cycle assessment

Mohammad Naji Nassajfar [a,*]. Jouni Havukainen[a]. Elaheh Aryapour[b]. Mariam Abdulkareem[a]. Mika Horttanainen[a]

[a] *Lappeenranta-Lahti University of Technology. School of Energy Systems. Department of Sustainability Science. P.O.Box 53851. Lappeenranta. Finland*

[b] *Stora Enso Oyj*

[*] *corresponding author (Email: mohammad.naji.nassajfar@lut.fi)*

*Table **S1-1**. Inventory data for Scenario 1 (ER).*

**Unit process**	**Amount**	**Unit**	**Process name**	**Reference**
ABS incineration	510	Kg	Acrylonitrile-butadiene-styrene (ABS) in waste incineration plant	Calculated
PS incineration	170	Kg	Polystyrene (PS) in a waste incineration plant	Calculated
PP incineration	150	Kg	Polypropylene (PP) in waste incineration plant	Calculated
PC incineration	160	Kg	Polycarbonate (PC) in waste incineration plant	Calculated
Avoided Electricity production	5845	MJ	Electricity grid mix of Finland	Sum of electricity production from ABS, PS, PC and PP incineration
Avoided heat production	10550	MJ	District heating grid mix of Finland	Sum of heat production from ABS, PS, PC and PP incineration

Table **S1-2**. Environmental impact of Scenario 1 (ER).

**Environmental Impact***	**Total**	**Incineration**	**Avoided Electricity**	**Avoided heat**
**Abiotic Depletion (ADP elements). Economic Reserve [**kg **Sb eq.]**	-2.19E-04	2.86E-05	-1.68E-04	-7.99E-05
**Abiotic Depletion (ADP elements). Reserve Base [**kg **Sb eq.]**	-1.20E-04	1.33E-05	-8.96E-05	-4.33E-05
**Abiotic Depletion (ADP elements) [**kg **Sb eq.]**	-9.60E-06	5.92E-05	-5.43E-05	-1.45E-05
**Abiotic Depletion (ADP fossil) [MJ]**	-1.01E+04	4.83E+02	-3.20E+03	-7.35E+03
**Acidification Potential (AP) [**kg **SO2 eq.]**	-2.26E+00	4.80E-01	-8.63E-01	-1.88E+00
**Eutrophication Potential (EP) [**kg **Phosphate eq.]**	-2.02E-01	9.53E-02	-1.00E-01	-1.97E-01
**Freshwater Aquatic Ecotoxicity Pot. (FAETP inf.) [**kg **DCB eq.]**	-1.75E+00	1.20E-01	-1.28E+00	-5.87E-01
**Global Warming Potential (GWP 100 years) [**kg **CO2 eq.]**	1.21E+03	2.29E+03	-3.31E+02	-7.50E+02
Global Warming Potential (GWP 100 years). excl biogenic carbon [kg CO2 eq.]	1.21E+03	2.29E+03	-3.29E+02	-7.47E+02
**Human Toxicity Potential (HTP inf.) [**kg **DCB eq.]**	-6.77E+01	9.96E+00	-3.04E+01	-4.73E+01
**Marine Aquatic Ecotoxicity Pot. (MAETP inf.) [**kg **DCB eq.]**	-5.73E+05	7.18E+04	-2.22E+05	-4.23E+05
**Ozone Layer Depletion Potential (ODP. steady state) [**kg **R11 eq.]**	-5.27E-13	2.92E-13	-7.22E-13	-9.67E-14
**Photochem. Ozone Creation Potential (POCP) [**kg **Ethene eq.]**	-1.91E-01	2.91E-02	-7.10E-02	-1.49E-01
**Terrestric Ecotoxicity Potential (TETP inf.) [**kg **DCB eq.]**	-1.18E+00	2.26E-01	-4.98E-01	-9.09E-01

* All the environmental impacts are in CML2001 - Aug. 2016 method.

*Table **S2-1**. Inventory data for Scenario 2 (MRC).*

Unit process	Amount	Unit	Details	Reference
Electricity Consumption				
Washing & Drying	1584	MJ	(1.98 MJ/kg)	Alston et al. (2011)
Crushing	133.3	MJ	(0.17 MJ/kg)	Sphera database
Extrusion	2152	MJ	(2.76 MJ/kg)	Sphera database
Total electricity consumption	3870	MJ		
Thermal energy consumption	335.4	MJ	(0.43 MJ/kg)	Sphera database
Water consumption	7.9	kg		Sphera database
Compressed air	15.6	Nm3	(0.02 Nm3/kg)	Sphera database
Lubricating oil (extrusion)	0.1	kg	(0.14 gr/kg)	Sphera database

*Table **S2-2**. Environmental impacts of Scenario 2 (MRC).*

Environmental Impact	Total	Crushing	Granulate production	Incineration	Washing & drying	XRF	Avoided-Electricity	Avoided-Heat	Avoided-material
(ADP elements). Economic Reserve [kg Sb eq.]	-2,21E-04	3.6E-06	7.0E-05	7.3E-06	4.3E-05	1.1E-05	-3,21E-05	-5,59E-05	-2.7E-04
(ADP elements). Reserve Base [kg Sb eq.]	-1,40E-04	1.9E-06	3.8E-05	3.4E-06	2.3E-05	6.1E-06	-1,71E-05	-3,24E-05	-1.6E-04
(ADP elements) [kg Sb eq.]	-7,19E-05	1.2E-06	2.1E-05	1.5E-05	1.4E-05	3.7E-06	-1,04E-05	-1,06E-05	-1.1E-04
(ADP fossil) [MJ]	-4,77E+03	6.9E+01	1.5E+03	1.2E+02	8.2E+02	2.2E+02	-610	-3,17E+03	-3.7E+03
(AP) [kg SO2 eq.]	-8,25E-01	1.9E-02	3.2E-01	1.2E-01	2.2E-01	5.8E-02	-0,164	-0,945	-4.6E-01
(EP) [kg Phosphate eq.]	-1,19E-01	2.2E-03	3.8E-02	2.4E-02	2.6E-02	6.8E-03	-0,019	-0,111	-8.6E-02
(FAETP inf.) [kg DCB eq.]	-6,95E-01	2.8E-02	4.6E-01	3.0E-02	3.3E-01	8.7E-02	-0,243	-0,727	-6.6E-01
(GWP 100 years) [kg CO2 eq.]	1,21E+02	7.2E+00	1.4E+02	5.8E+02	8.5E+01	2.2E+01	-63	-287	-3.6E+02
(GWP 100 years). excl biogenic carbon [kg CO2 eq.]	1,22E+02	7.1E+00	1.4E+02	5.8E+02	8.5E+01	2.2E+01	-62,7	-285	-3.6E+02
(HTP inf.) [kg DCB eq.]	-2,03E+01	6.6E-01	1.1E+01	2.5E+00	7.8E+00	2.1E+00	-5,8	-27,6	-1.1E+01
(MAETP inf.) [kg DCB eq.]	-1,63E+05	4.8E+03	7.9E+04	1.8E+04	5.7E+04	1.5E+04	-4,23E+04	-2,70E+05	-2.4E+04
(ODP. steady state) [kg R11 eq.]	-5,87E-12	1.6E-14	2.6E-13	7.4E-14	1.9E-13	4.9E-14	-1,38E-13	-5,52E-14	-6.3E-12
(POCP) [kg Ethene eq.]	-7,37E-02	1.5E-03	2.7E-02	7.4E-03	1.8E-02	4.8E-03	-0,0135	-0,0824	-3.6E-02

*Table **S3-1**. Inventory data for Scenario 3 (MRS).*

**Unit process**	**Amount**	**Unit**	**Details**	**Reference**
Washing & Drying	1584	MJ		
Crushing	134.64	MJ	(2.76 MJ/kg)	Sphera database
Air Separation	8.69	kg		Sphera database
Plastic Extrusion	2180.4	Nm3	(2.76 MJ/kg)	Sphera database
Pelletization	39.5	kg	(0.05 MJ/kg)	Sphera database

Table ***S3-2***. *Environmental impacts for Scenario 3 (MRS).*

**Environmental Impact**	**Total**	**Incineration**	**Granulate production**	**NIR**	**washing & drying**	**XRF**	**Avoided-Electricity**	**Avoided-Heat**	**Air separation**	**Avoided-material**
(ADP elements). Economic Reserve [kg Sb eq.]	-9,74E-04	7.48E-06	7.25E-05	8.94E-06	4.33E-05	1.14E-05	-3,21E-05	-5,59E-05	2.35E-07	-0.00103
(ADP elements). Reserve Base [kg Sb eq.]	-5,85E-04	3.47E-06	3.93E-05	4.76E-06	2.30E-05	6.07E-06	-1,71E-05	-3,24E-05	1.25E-07	-0.00061
(ADP elements) [kg Sb eq.]	-1,25E-03	1.55E-05	2.19E-05	2.88E-06	1.40E-05	3.68E-06	-1,04E-05	-1,06E-05	7.60E-08	-0.00129
(ADP fossil) [MJ]	-3,72E+04	1.26E+02	1.55E+03	170	823	217	-610	-3,17E+03	4.48	-3.63E+04
(AP) [kg SO2 eq.]	-2,01E+00	1.26E-01	0.329	0.0458	0.222	0.0584	-0,164	-0,945	0.00121	-1.68
(EP) [kg Phosphate eq.]	-3,48E-01	2.49E-02	0.0397	0.00531	0.0257	0.00677	-0,019	-0,111	0.00014	-0.32
(FAETP inf.) [kg DCB eq.]	-1,73E+01	3.12E-02	0.48	0.0678	0.328	0.0865	-0,243	-0,727	0.00179	-17.3
(GWP 100 years) [kg CO2 eq.]	-7,52E+02	5.99E+02	143	17.6	85	22.4	-63	-287	0.462	-1.27E+03
(GWP 100 years). excl biogenic carbon [kg CO2 eq.]	-7,52E+02	5.99E+02	142	17.5	84.6	22.3	-62,7	-285	0.46	-1.27E+03
(HTP inf.) [kg DCB eq.]	-7,73E+02	2.60E+00	11.5	1.62	7.83	2.06	-5,8	-27,6	0.0426	-765
(MAETP inf.) [kg DCB eq.]	-1,78E+05	1.88E+04	8.14E+04	1.18E+04	5.71E+04	1.50E+04	-4,23E+04	-2,70E+05	311	-5.00E+04
(ODP. steady state) [kg R11 eq.]	-7,17E-07	7.63E-14	2.66E-13	3.83E-14	1.86E-13	4.89E-14	-1,38E-13	-5,52E-14	1.01E-15	-7.17E-07
(POCP) [kg Ethene eq.]	-3,05E-01	7.61E-03	0.0276	0.00377	0.0182	0.0048	-0,0135	-0,0824	9.93E-05	-0.271
Terrestric Ecotoxicity Potential (TETP inf.) [kg DCB eq.]	-4,44E+00	5.90E-02	0.236	0.0265	0.128	0.0337	-3,21E-05	-5,59E-05	0.000697	-4.23

*Table **S4-1**. Inventory data for Scenario 4 (CR).*

**Electricity & Heat Consumption**	**Value**	**Unit**	**Details**
Washing & Drying	1584	MJ	
Filtration	41	MJ	(0.18 MJ/kg)
Chemical Distillation	32	MJ	(0.18 MJ/kg)
Polymerization electricity demand	99	MJ	(2.1 MJ/kg)
Polymerization heat demand	56	MJ	(1.2 MJ/kg)
Plastic extrusion electricity demand	121	MJ	(2.76 MJ/kg)
Plastic extrusion heat demand	18.9	MJ	(0.43 MJ/kg)
Pelletization	2.2	MJ	(0.05 MJ/kg)
Total Electricity consumption	296	MJ	
Total Electricity consumption	8985	MJ	
Pyrolysis Heat demand (Internal resource)	8910	MJ	Obtained from incineration of pyrolysis products

*Table **S4-2**. Environmental impacts for Scenario 4 (CR).*

**Environmental Impact**	**Total**	**Granulate production**	**Incineration ABS**	**Pyrolysis**	**washing & drying**	**XRF**	**Avoided-Electricity**	**Avoide d-Heat**	**Avoided Electricity (Pyrolysis)**	**Avoided-heat (Pyrolysis)**	**Avoided material**
(ADP elements). Economic Reserve [kg Sb eq.]	-2,66E-04	2.78E-05	7.25E-06	3.92E-05	4.33E-05	1.14E-05	-3,21E-05	-5,59E-05	-1.68E-05	-0.00023	-5.77E-05
(ADP elements). Reserve Base [kg Sb eq.]	-1,63E-04	1.19E-05	3.36E-06	2.00E-05	2.30E-05	6.07E-06	-1,71E-05	-3,24E-05	-8.48E-06	-0.00013	-3.54E-05
(ADP elements) [kg Sb eq.]	-2,70E-07	7.36E-06	1.50E-05	4.74E-05	1.40E-05	3.68E-06	-1,04E-05	-1,06E-05	-4.31E-06	-4.40E-05	-1.84E-05
(ADP fossil) [MJ]	-1,81E+04	145	122	809	823	217	-610	-3,17E+03	-39.7	-1.31E+04	-3.27E+03
(AP) [kg SO2 eq.]	-4,31E+00	0.0595	0.122	0.379	0.222	0.0584	-0,164	-0,945	-0.0152	-3.91	-0.118
(EP) [kg Phosphate eq.]	-4,67E-01	0.00961	0.0241	0.0754	0.0257	0.00677	-0,019	-0,111	-0.00274	-0.459	-0.0165
(FAETP inf.) [kg DCB eq.]	-4,30E+00	0.0859	0.0303	0.116	0.328	0.0865	-0,243	-0,727	-0.0357	-3.01	-0.931
(GWP 100 years) [kg CO2 eq.]	7,79E+02	14.9	581	1.72E+03	85	22.4	-63	-287	-3.95	-1.19E+03	-100
(GWP 100 years). excl biogenic carbon [kg CO2 eq.]	7,91E+02	14.9	581	1.72E+03	84.6	22.3	-62,7	-285	-3.95	-1.18E+03	-99.9
(HTP inf.) [kg DCB eq.]	-1,30E+02	1.22	2.52	8.06	7.83	2.06	-5,8	-27,6	-0.317	-114	-4.18
(MAETP inf.) [kg DCB eq.]	-1,28E+06	8.14E+03	1.82E+04	5.48E+04	5.71E+04	1.50E+04	-4,23E+04	-2,70E+05	-239	-1.12E+06	-3.45E+03
(ODP. steady state) [kg R11 eq.]	-2,85E-13	3.27E-14	7.40E-14	2.73E-13	1.86E-13	4.89E-14	-1,38E-13	-5,52E-14	-1.68E-13	-2.28E-13	-3.10E-13
(POCP) [kg Ethene eq.]	-4,02E-01	0.00341	0.00738	0.024	0.0182	0.0048	-0,0135	-0,0824	-0.00061	-0.341	-0.022
(TETP inf.) [kg DCB eq.]	-2,93E+00	0.0245	0.0572	0.232	0.128	0.0337	-3,21E-05	-5,59E-05	-0.00717	-2.46	-0.248
